# Effects of rAmb a 1-Loaded PLGA-PEG Nanoparticles in a Murine Model of Allergic Conjunctivitis

**DOI:** 10.3390/molecules27030598

**Published:** 2022-01-18

**Authors:** Hui Cao, Ling Liu, Junyi Wang, Miao Gong, Ruyi Yuan, Jiahua Lu, Xiaojun Xiao, Xiaoyu Liu

**Affiliations:** 1 State Key Laboratory of Respiratory Disease for Allergy at Shenzhen University, Shenzhen Key Laboratory of Allergy & Immunology, Shenzhen University School of Medicine, Shenzhen 518061, China; huiencao@163.com (H.C.); liuling629917493@163.com (L.L.); wjy520wjy@163.com (J.W.); yry1230@126.com (R.Y.); asssixgod@163.com (J.L.); 2 Sichuan Vocational College of Health and Rehabilitation, Zigong 643000, China; 3 Laboratory of Allergy and Precision Medicine, Chengdu Institute of Respiratory Health, The Third People’s Hospital of Chengdu, Chengdu 610031, China; 4 Department of Respirology & Allergy, Third Affiliated Hospital of Shenzhen University, Shenzhen 518020, China; gmiao1992@163.com

**Keywords:** poly(DL-lactide-co-glycolide)-polyethylene glycol (PLGA-PEG), allergic conjunctivitis, allergen, Amb a 1, allergen-specific immunotherapy

## Abstract

*Ambrosia artemisiifolia* (Amb a) contains many allergens. Allergic conjunctivitis caused by *Ambrosia artemisiifolia* and its related allergen-specific immunotherapy (AIT) are seldom studied at present. poly(DL-lactide-co-glycolide)-polyethylene glycol (PLGA-PEG) is a very good nano-carrier, which has been applied in the medical field. In this context, we studied the immunotherapy effect and potential mechanism of recombinant Amb a 1 (rAmb a 1)-loaded PLGA-PEG nanoparticles. A mouse allergic conjunctivitis model was established with *Ambrosia artemisiifolia* crude extract, and the nanoparticles were used for AIT through direct observation of conjunctival tissue, degranulation of mast cells in conjunctival tissue, serum-specific antibodies, cytokines and other assessment models. The treatment of nanoparticles enhanced the secretion of T-helper 1 (Th1) cytokine Interferon-gama (IFN-γ) and the production of immunoglobulin G (IgG)2a (IgG2a), inhibited the secretion of T-helper 2 (Th2) cytokine Interleukin (IL)-13 and IL-4 and the level of IgE. Especially, degranulation of mast cells and expression of mast cell protease-1 (MCP-1) in conjunctival tissue was reduced significantly. In this study, we proved that the nanoparticles prepared by rAmb a 1 and PLGA-PEG have an immunotherapy effect on allergic conjunctivitis in mice.

## 1. Introduction

Allergic conjunctivitis is one of the most common ocular allergic diseases; approximately 10% to 30% of the general population has ocular allergic symptoms [1]. The pathogenesis of allergic conjunctivitis is complex and can be divided into early response (EPR) and late response (LPR), and EPR is mainly an inflammatory reaction mediated by allergen-specific IgE [2]. The infiltration of eosinophils, the activation of T cells, and the expression and secretion of some cytokines, chemokines, and adhesion factors also participate in its occurrence and development [3,4]. The main symptoms of allergic conjunctivitis include conjunctival congestion, swelling, tears, photophobia, etc [5,6]. Pollen is one of the most common air allergens, which has seasonal and spatial differences and can induce allergic diseases such as allergic rhinitis, bronchial asthma and allergic conjunctivitis [7,8]. Allergies to pollen are also called hay fever, and studies have shown that at least 10% of the global population is allergic to pollen [8]. *Ambrosia artemisiifolia* originates from North America and has been widely distributed in most parts of the world now. Its pollen is an important airborne allergen. Amb a 1 is a 38 kDa non-glycosylated acidic protein with a positive rate of over 90% in patients allergic to *Ambrosia artemisiifolia* [9].

Allergen-specific immunotherapy (AIT) is the only cause-specific therapy for allergic diseases. The method is to stimulate allergic patients from a low dose to a high dose with allergens, and finally to cause patients to not respond to allergen stimulation, that is, to achieve immune tolerance [10]. AIT has a history of over 100 years, but at present, most vaccine products used clinically are crude extracts of allergens [11,12]. Due to the shortcomings of these crude extracts, such as complex components, large differences between batches, etc., they often cause patients to experience adverse reactions and even treatment failures in the treatment process [13]. Therefore, it is necessary to develop a new AIT vaccine to solve the existing problems. PLGA (polylactic acid–glycolic acid) is a non-toxic polymer material certified by the FDA (the United States Food and Drug Administration), which is biodegradable and can be used as an auxiliary material for clinical drugs [14]. PEG (Polyethylene Glycol) is also a polyether polymer material certified by the FDA with long internal circulation and a high level of biological safety. PEG has two affinity characteristics of dissolving in water and organic solvents, which can supplement the hydrophobicity of PLGA [15]. Nanoparticles prepared by embedding PEG in PLGA-carrier materials combine the advantages of a controlled-release mode, biodegradability, and biocompatibility, and they can be used as drug-delivery carriers with the advantages of safety, high stability, and a prolonged period of immunity [16].

In this study, we constructed rAmb a 1-loaded PLGA-PEG nanoparticles for the treatment of allergic conjunctivitis caused by a crude extract of *Ambrosia artemisiifolia*, and we evaluated their effect and potential mechanism.

## 2. Results

### 2.1. Characterization of rAmb a 1-Loaded PLGA-PEG Nanoparticles

**Figure 1:**
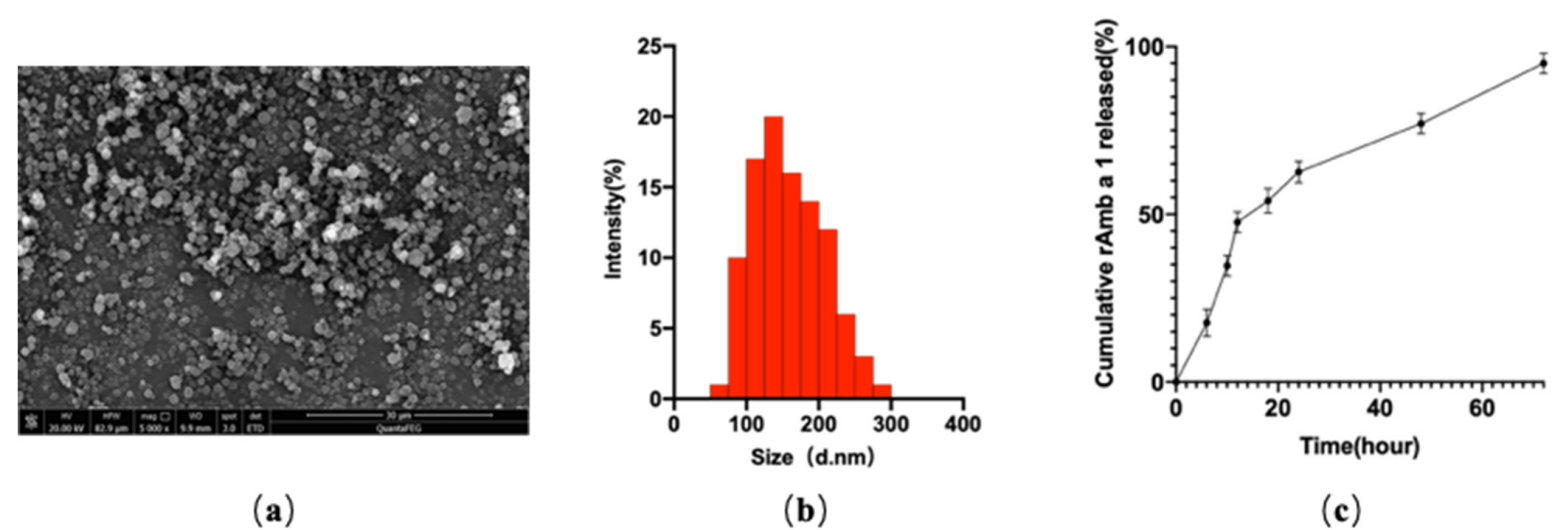
(**a**) Field emission scanning electron microscopic image; (**b**) dynamic light-scattering spectra; (**c**) in vitro cumulative protein release of rAmb a 1-loaded PLGA-PEG nanoparticles. Abbreviations: PLGA-PEG, poly(DL-lactide-co-glycolide)-polyethylene glycol; rAmb a 1, recombinant Amb a1.

The dynamic light-scattering observation shows that the nanoparticles are about 80–250 nm in diameter (Figure 1a,b). It also shows a negative surface charge around −22 mV, which was stable enough to remain in the body. The zeta potential of nanoparticles can cause the nanoparticles to form electrostatic repulsion, cause the suspension to exist stably, and it is also beneficial to the interaction between nanoparticles and the cell membrane in vivo [17]. The protein *LC* (loading content) of the rAmb a 1-loaded PLGA nanoparticles was 13.72%, and *EE* (encapsulation efficiency) was 88.21%. rAmb a 1-loaded PLGA-PEG nanoparticles also show a good sustained-release effect (Figure 1c).

### 2.2. SDS-PAGE and Western Blotting of rAmb a 1-Loaded PLGA–PEG Nanoparticles

**Figure 2:**
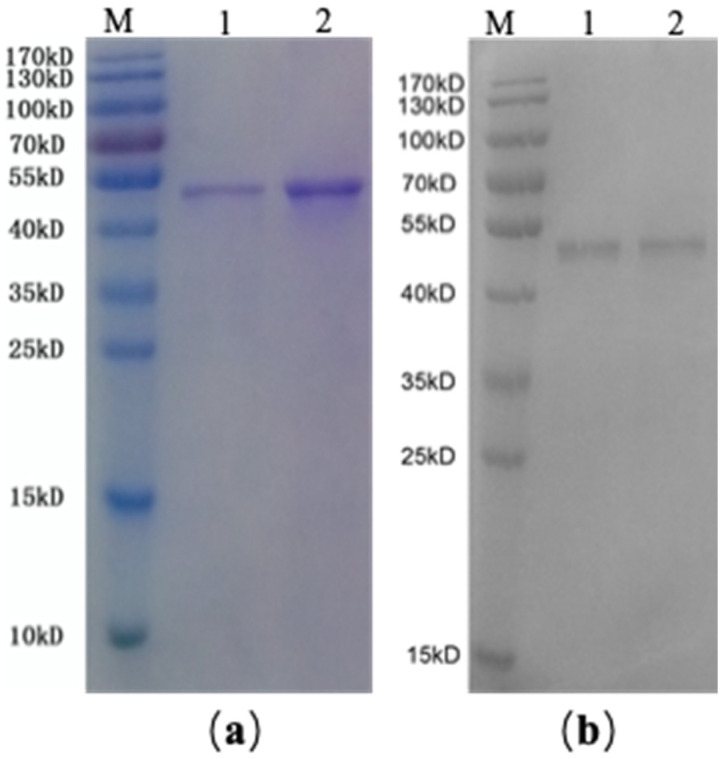
(**a**) SDS-PAGE analysis of rAmb a 1-loaded PLGA-PEG and rAmb a 1, M: protein marker, 1: rAmb a 1-PLGA-PEG, 2: rAmb a 1; (**b**) Western blot of rAmb a 1-loaded PLGA-PEG and rAmb a 1 with serum from an *Ambrosia artemisiifolia*-allergic patient, M: protein marker, 1: rAmb a 1-PLGA-PEG, 2: rAmb a 1. Abbreviations: SDS-PAGE, sodium dodecyl sulfate-polyacrylamide gel electrophoresis; PLGA-PEG, poly(DL-lactide-co-glycolide)-polyethylene glycol; rAmb a 1, recombinant Amb a 1.

SDS-PAGE (sodium dodecyl sulfate-polyacrylamide gel electrophoresis) showed that the molecular weight of PLGA-PEG-coated rAmb a 1 was the same as rAmb a 1, which indicated that, when PLGA-PEG coated rAmb a 1, the properties of rAmb a 1 did not change (Figure 2a). In addition, it was found that specific IgE in serum was combined with PLGA-PEG + rAmb a 1 by Western blot (Figure 2b), which indicated that PLGA-PEG does not change the immunological characteristics of rAmb a 1.

### 2.3. Evaluation of Ocular Signs

**Figure 3:**
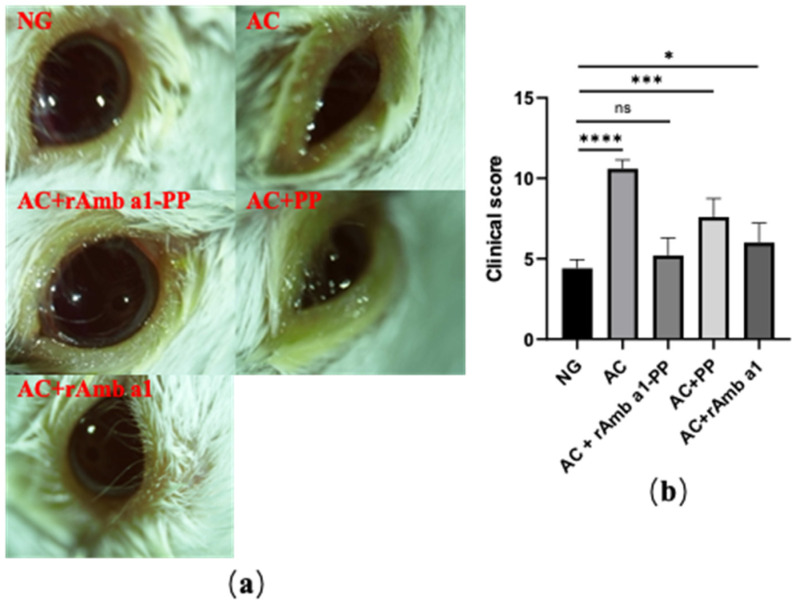
(**a**) Ocular signs of allergic conjunctivitis in mice in each group; (**b**) eye feature scores of mice in each group. The data are shown as mean ± SD from five individual mice (**** *p* < 0.0001, *** *p* < 0.001, * *p* < 0.05, ns: no significant difference). Abbreviations: NG, naive group; AC, allergic conjunctivitis group; AC + rAmb a 1-PP, allergic conjunctivitis + rAmb a 1-PLGA-PEG treatment group; AC + PP, allergic conjunctivitis + PLGA-PEG treatment group; AC + rAmb a 1, allergic conjunctivitis + rAmb a 1 treatment group.

The early response (EPR) of allergic conjunctivitis in mice in each group was observed and scored (Figure 3). The results showed the eye symptoms of mice in the allergic conjunctivitis group (AC), allergic conjunctivitis + rAmb a 1 treatment group (AC + rAmb a 1) and allergic conjunctivitis + PLGA-PEG treatment group (AC + PLGA-PEG) groups were more serious, and the score was also higher than that in naive group (NG). Compared with the NG, there was no significant change in the characteristics of EPR in the allergic conjunctivitis + rAmb a 1-PLGA-PEG treatment group (AC + rAmb a 1-PLGA-PEG). The above results indicate that rAmb a 1-PLGA-PEG can effectively relieve the symptoms of allergic conjunctivitis.

### 2.4. Analysis of Histological Examination

**Figure 4:**
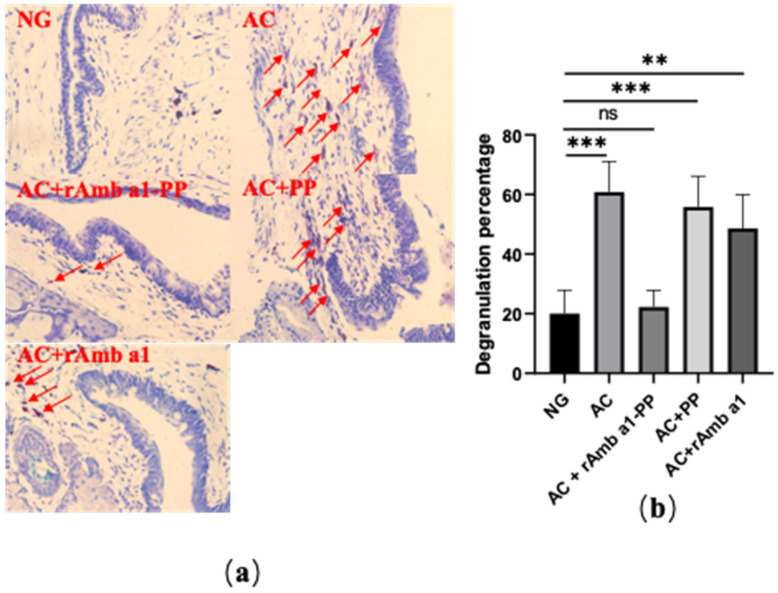
(**a**) Formalin-fixed conjunctival tissue section with mast cells stained with toluidine blue; (**b**) degranulation rate of mast cells in mice in each group. The data are shown as mean ± SD from four individual mice (*** *p* < 0.001, ** *p* < 0.01, ns: no significant difference). Abbreviations: NG, naive group; AC, allergic conjunctivitis group; AC + rAmb a 1-PP, allergic conjunctivitis + rAmb a 1-PLGA-PEG treatment group; AC + PP, allergic conjunctivitis + PLGA-PEG treatment group; AC + rAmb a 1, allergic conjunctivitis + rAmb a 1 treatment group.

We sliced the conjunctival tissue of the mice with paraffin and stained the mast cells with toluidine blue (Figure 4a). There was no significant difference in the degranulation rate of the mast cells in the conjunctival tissue between the naive group and the AC + rAmb a 1-PLGA-PEG group (*p* > 0.05). In the other three groups, the degranulation rate of the mast cells was significantly higher than that in the naive group (*** *p* < 0.001, ** *p* < 0.01) (Figure 4b).

### 2.5. Specific IgE and IgG2a in Serum

**Figure 5:**
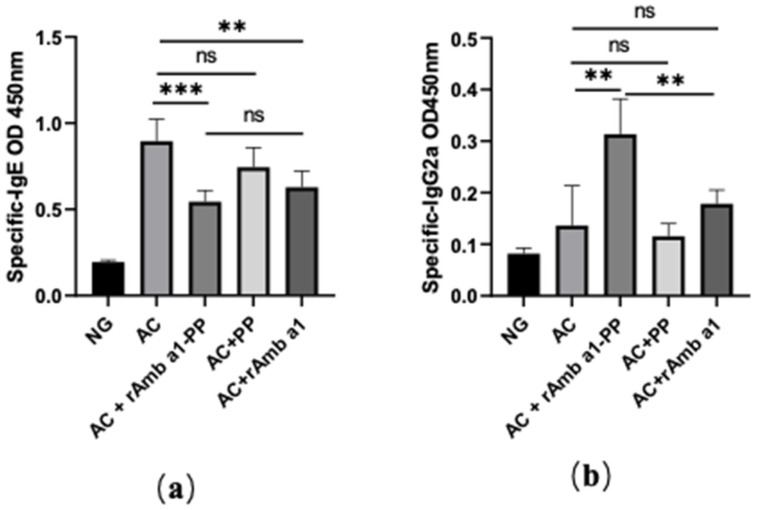
Serum-specific IgE (**a**) and IgG2a (**b**) in mice in each group. The data are shown as mean ± SD from five individual mice (*** *p* < 0.001, ** *p* < 0.01, ns: no significant difference). Abbreviations: NG, naive group; AC, allergic conjunctivitis group; AC + rAmb a 1-PP, allergic conjunctivitis + rAmb a 1-PLGA-PEG treatment group; AC + PP, allergic conjunctivitis + PLGA-PEG treatment group; AC + rAmb a 1, allergic conjunctivitis + rAmb a 1 treatment group.

Aiming at evaluating the effect of treatment with rAmb a 1-PLGA-PEG nanoparticles on systemic antibody responses, the levels of Amb a-specific antibody (IgG2a, IgE) in serum were measured (Figure 5). The Amb a-sensitized mice developed Th2-biased antibody responses characterized by high levels of specific IgE, whereas the treatment with rAmb a 1-PLGA-PEG nanoparticles reduced the specific IgE and increased the specific IgG2a levels compared to the other three treatment groups and the positive group. Treatment with rAmb a 1 alone can also reduce the specific IgE and increase the IgG2a level, but obviously, the treatment effect is not as good as that of rAmb a 1-PLGA-PEG. The results also showed that PLGA-PEG treatment alone had no therapeutic effect on mice.

### 2.6. rAmb a 1-PLGA-PEG Nanoparticles Enhances the Th1/Th2 Ratio

**Figure 6:**
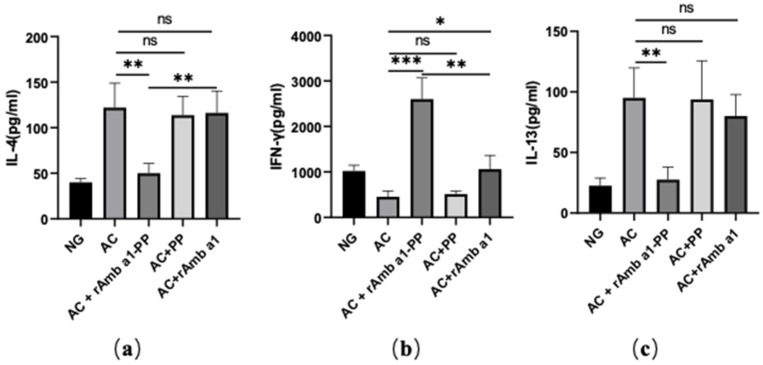
IL-4 (**a**), IFN-γ (**b**) and IL-13 (**c**) in each group. Cytokine production in spleen cell cultures. The data are shown as mean ± SD from five individual mice (*** *p* < 0.001, ** *p* < 0.01, * *p* < 0.05. ns: no significant difference). Abbreviations: NG, naive group; AC, allergic conjunctivitis group; AC + rAmb a 1-PP, allergic conjunctivitis + rAmb a 1-PLGA-PEG treatment group; AC + PP, allergic conjunctivitis + PLGA-PEG treatment group; AC + rAmb a 1, allergic conjunctivitis + rAmb a 1 treatment group.

IL-4 and IL-13 are typical Th 2 cytokines; the former influences the secretion of IgE, while the latter can aggravate allergic reactions [18]. IFN-γ is a Th1 cytokine, and the main mechanism of allergen-specific immunotherapy is the transfer of Th2 cell response to Th1 cell response [19]. Therefore, the levels of cytokines in the Amb a-stimulated spleen were measured (Figure 6). Amb a-sensitization can increase the levels of IL-4 and IL-13 and reduce the level of IFN-γ. After treatment with the rAmb a 1-PLGA-PEG nanoparticles, the levels of IL-4 and IL-13 decreased, while the level of IFN-γ increased, but the cytokines in other treatment groups did not change significantly, which indicated that the T-cell response of mice after treatment with rAmb a 1-PLGA-PEG nanoparticles changed from Th2 to Th1.

### 2.7. rAmb a 1-PLGA-PEG Nanoparticles Inhibits Mast Cell Degranulation

**Figure 7:**
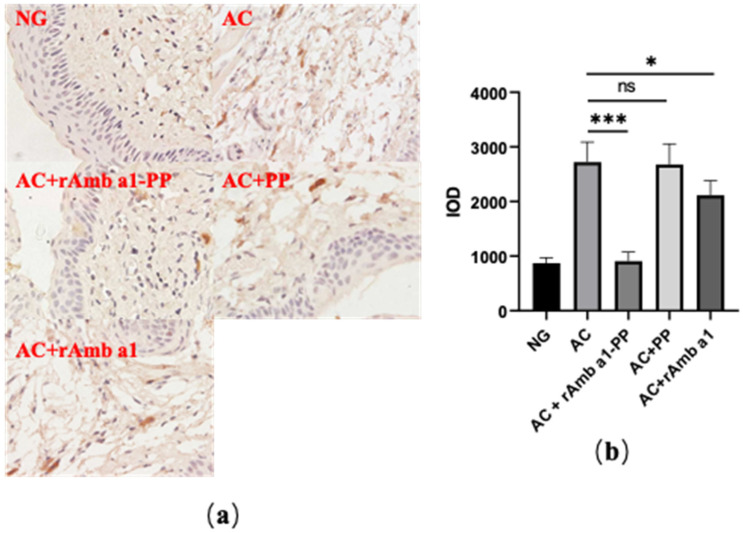
(**a**) Immunohistochemical method to stain mMCP-1 in paraffin section of mice conjunctival tissue; (**b**) IOD value in each group. The data are shown as mean ± SD from four individual mice (*** *p* < 0.001, * *p* < 0.05, ns: no significant difference). Abbreviations: NG, naive group; AC, allergic conjunctivitis group; AC + rAmb a 1-PP, allergic conjunctivitis + rAmb a 1-PLGA-PEG treatment group; AC + PP, allergic conjunctivitis + PLGA-PEG treatment group; AC + rAmb a 1, allergic conjunctivitis + rAmb a 1 treatment group; IOD, integrated optical density; mMCP-1, mouse mast cell proteinase-1.

Mouse mast cell proteinase-1 (mMCP-1) can be used as a marker for degranulation of mast cells [20]. Therefore, we used an immunohistochemical method to stain mMCP-1 in a paraffin section of mice conjunctival tissue and calculated the integrated optical density (IOD) value of mMCP-1 with ImagePlus software (Figure 7). After treatment with the rAmb a 1-PLGA-PEG nanoparticles, the IOD value of mMCP-1 decreased significantly. The IOD value of mMCP-1 can also be reduced to a certain extent by rAmb a 1 alone, but the effect is far less than that of rAmb a 1-PLGA-PEG nanoparticles.

## 3. Discussion

Based on its safety and slow-release properties, PLGA-PEG has been demonstrated to be an effective nanodrug carrier [21]. In light of this, the recombinant allergen protein was encapsulated by PLGA-PEG and slowly released into allergic patients in order to induce allergen specific immune tolerance. It has become a promising concept of nanovaccine intervention against Type I allergy [22]. Therefore, we constructed rAmb a 1-PLGA-PEG nanoparticles and evaluated their AIT potential in a mouse allergic conjunctivitis model with rAmb a 1 as the allergen.

PLGA-PEG nanoparticles can be developed as nanodrug carriers because their particle size has reached the nanometer level, and the encapsulated drugs can be slowly released. Previous studies have shown that the particle size of drug-loaded PLGA-PEG nanoparticles is between 50–250 nm [23,24]. In this study, the particle size of rAmb a 1-loaded PLGA-PEG nanoparticles is between 50–300 nm, and most of them are between 100–200 nm, which is consistent with other researchers’ results. The slow release of nanomaterials can increase the interaction time between antigen and immune cells [25]. In terms of their slow-release characteristics, the 72 h release rate of curcumin-loaded PLGA-PEG nanoparticles prepared by researcher Sheng Nuofan and his colleagues was 78.01 ±  1.55% [23], while the 72 h release rate of nanoparticles prepared by us was 95 ± 3%, which may be due to the characteristics of the encapsulated drug.

Allergen-specific IgE mediates Type I hypersensitivity, which is also a sign for the clinical diagnosis of allergic diseases [26]. This study showed that injection of rAmb a 1-loaded PLGA-PEG nanoparticles could inhibit the specific allergic reaction of Ambrosia artemisiifolia and the specific IgE level decreased in the serum. In the process of allergen-specific immunotherapy, the highly specific IgG2a antibody is induced to transfer the Th2 reaction to Th1, thus inhibiting the allergic reaction [27]. In this study, the level of IgG2a antibody in the treatment group of rAmb a 1-loaded PLGA-PEG nanoparticles was significantly increased. A high concentration of IgG2a antibodies can also specifically bind to allergens, thus preventing the combination of IgE and *Ambrosia artemisiifolia* allergens and alleviating allergic inflammation [28]. Therefore, PLGA-PEG nanoparticles coated with allergens should be considered as a strategy for the treatment of allergic diseases.

As is universally accepted, the internal profile of the Th1/Th2 reaction determines the direction of antibody response. IL-4 is a Th2 cytokine, which plays an important role in promoting B-cell proliferation and stimulating B cells to secrete IgE [18]. In the supernatant of the spleen-cell culture in vitro, the secretion of IL-4 in mice treated with rAmb a 1-loaded PLGA-PEG nanoparticles was significantly inhibited. On the contrary, with the Th1 cytokine, the level of IFN-γ remarkably improved. These results are consistent with our previous studies. Similar to IL-4, IL-13 plays an important role in the induction and maintenance of Th2 cellular immune response [18,29]. The decrease in IL-13 in the rAmb a1-loaded PLGA-PEG nanoparticle treatment group also shows that the ratio of Th1/Th2 increases and the allergic reaction is reduced.

On the whole, treatment with allergen-loaded PLGA-PEG nanoparticles after sensitization exerted beneficial effects on the suppression of allergic responses, suggesting that allergen-loaded PLGA-PEG nanoparticles would be an ideal vaccine for the treatment of allergic diseases in the future. This therapeutic effect is mainly due to the increase in the Th1/Th2 ratio, the change in IgE and IgG2a antibody levels, and the reduction in mast-cell degranulation. In addition to the increase in the Th1/Th2 ratio, the increase in regulatory T cells (Tregs) and the epigenetic changes in T cells will also influence the effect of allergen-specific immunotherapy [30]. In order to understand the mechanism more deeply, it is necessary to further confirm the effect of allergen-loaded PLGA-PEG nanoparticles on effector T cells, Tregs, and epigenetic changes in T cells [31,32].

## 4. Materials and Methods

### 4.1. Animals and Materials

Male BALB/c mice, 6 weeks old, were purchased from the Medical Experimental Animal Center of Guangdong Province (Guangzhou, China) and fed in a specific pathogen-free-grade breeding room. PLGA–PEG and polyvinyl alcohol were purchased from Sigma-Aldrich (St. Louis, MO, USA). Dichloromethane was purchased from J&K Chemical Co, Ltd. (Beijing, China). HPR-labeled goat anti-mouse IgG1, IgG2a, IgE, and HPR-labeled goat anti-human IgE were purchased from Southern Biotechnology Associates Inc. (Birmingham, AL, USA). Mouse IL-4, IL-13, and IFN-γ ELISA kits were purchased from BioLegend (San Diego, CA, USA). Anti-MCP1 antibody (ab25124) was purchased from Abcam (Cambridge, MA, USA). Goat anti-rabbit IgG antibody was purchased from Cell Signaling Company (Beverley, MA, USA).

### 4.2. Expression and Purification of rAmb a 1

The plasmid-containing Amb a 1 gene was induced by 0.1 mM IPTG (isopropyl-beta-D-thiogalactopyranoside) to express rAmb a 1 in BL21 (DE3) Escherichia coli. The rAmb a 1 was purified with an Ni^2+^ affinity chromatography column (data not shown). Dialysis and freeze-drying were performed on the eluent-containing protein.

### 4.3. Preparation of rAmb a 1-Loaded PLGA–PEG Nanoparticles

The preparation of rAmb a 1-loaded PLGA–PEG nanoparticles was performed using a method of double emulsion solvent-evaporation [33]. One hundred microliters of rAmb a 1 (20 mg/mL) was added to dichloromethane dissolved with PLGA–PEG (50 mg). After ultrasonic treatment for 1 min (40 w), 2 mL of 2% polyvinyl alcohol (PVA) was put into the initial emulsion. After ultrasonic treatment for 1 min, the secondary emulsion was transferred to 50 mL of sterile deionized water, stirred at room temperature for 4 h, and volatile organic solvents were removed by volatilization. The particles were collected by centrifugation at a speed of 10,000 rpm for 10 min and washed 3 times with sterile distilled water. After freeze-drying, the preparation can be stored at 4 °C.

### 4.4. Characterization of rAmb a 1-Loaded PLGA-PEG Nanoparticles

Size and zeta potential: in order to determine the nanoparticle size and size distribution, about 0.2 mg of nanoparticles were diluted into 1 mL of distilled water, ultrasonically treated at 4 °C for 30 s, and measured using dynamic light scattering.

Surface morphology: after they were fixed on the stub with double-sided sticky tape, the nanoparticles were coated with a platinum layer using an automatic fine platinum coater (JFC-1300, JEOL) for 1 min, and then an observation was taken using field emission scanning electron microscopy (FESEM).

Protein loading and encapsulation efficiency: the BCA assay kit was used to detect the protein-loading content (*LC*) and encapsulation efficiency (*EE*) of the rAmb a 1-loaded PLGA-PEG nanoparticles [34]. In order to determine the protein concentration, 5 mg of nanoparticles were added to 2 mL aqueous solution of 0.05 mol/L NaOH/1% (*w*/*v*) SDS mixture and oscillated in a thermostated oscillator for 24 h to dissolve them thoroughly. The following equations were applied to calculate the protein *LC* and the *EE* of nanoparticles:

$$LC\left(\%\right)=\frac{protein\;in\;nanoparticles\left(weight\right)}{nanoparticles\left(weight\right)}\times 100$$

$$EE\left(\%\right)=\frac{protein\;in\;nanoparticles\left(weight\right)}{feeding\;protein\left(weight\right)}\times 100$$

Release study in vitro: 20 mg of rAmb a 1-loaded PLGA-PEG nanoparticles and 5 mL of PBS were cultured in an oscillating water bath at 37 °C at 120 rpm. A 1 mL sample was collected by centrifugation at a speed of 20,000 rpm for 30 min and replaced with PBS at appropriate time intervals. The bicinchoninic acid assay method was used to detect the fraction of protein released in samples.

Western blotting: the sample (rAmb a 1 and rAmb a 1-loaded PLGA-PEG nanoparticles) after SDS-PAGE gel electrophoresis was transferred to polyvinylidene fluoride (PVDF), and the PVDF membrane was blocked with 5% non-fat milk powder in TBST (20 mM Tris, 150 mM NaCl, 0.1% Tween-20, pH 7.4) for one hour at room temperature. To investigate the specific interaction between the protein and the serum of an Ambrosia artemisiifolia-allergic patient, the PVDF membrane was incubated overnight with a five-fold dilution of the patient serum (1:5 in 5% non-fat milk powder, TBST) at 2–8 °C. After it had been washed with TBST three times (5 min each), the PVDF membrane was incubated with HRP-labeled goat anti-human IgE antibody (1:2000 in 5% non-fat milk powder, TBST) at room temperature for one hour. After it had been washed with TBST three times (5 min each), the results were obtained by staining with ECL and analyzing it using a Chemiluminescence imaging system (GuangYi Biotechnology, Guangzhou, China).

### 4.5. Animal Sensitization and Specific Immunotherapy

**Figure 8:**
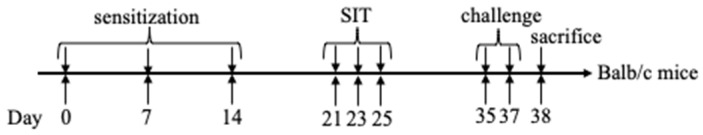
Experimental design.

The BALB/c mice were randomly divided into 5 groups with 6 mice in each group, named naive group (NG), allergic conjunctivitis group (AC), allergic conjunctivitis + rAmb a 1-PLGA-PEG treatment group (AC + rAmb a 1-PLGA-PEG), allergic conjunctivitis + rAmb a 1 treatment group (AC + rAmb a 1) and allergic conjunctivitis + PLGA-PEG treatment group (AC + PLGA-PEG), respectively. The animal sensitization model and SIT treatment plan were established based on the reference method with modifications (Figure 8). On days 0, 7, and 14, the mixture of Ambrosia artemisiifolia pollen crude protein (20 μg/50 μL) and 50 μL (2 mg) aluminum adjuvant (Sigma–Aldrich, St. Louis, MO, USA) was injected into the abdominal cavity of all of the groups except the naive group. From 21 to 28 days, the mice in the AC + rAmb a 1-PLGA-PEG, AC + rAmb a 1 and AC + PLGA-PEG groups were given nanoparticles (containing 50 μg rAmb a 1), 50 μg rAmb a 1, and an equivalent amount of PLGA-PEG, respectively, by subcutaneous (s.c.) injection every other day, three times. The naive group was treated with the same dose of physiological saline. The mice were challenged with the eye drops of 50 μg Ambrosia artemisiifolia pollen on day 35 and 37.

Twenty minutes after the last local challenge, the clinical reactions were checked and graded according to the criteria described in a previous report. The cumulative clinical score was calculated by evaluating chemosis, conjunctival redness, lid edema, and tearing, graded on a scale ranging from 0 to 3.

### 4.6. Histologic Evaluation

The mice were sacrificed and the conjunctival tissues were fixed in 4% paraformaldehyde, dehydrated, waxed, embedded in paraffin, sliced in series, and stained with toluidine blue. The number of mast cells in three consecutive sections from each eye were counted under a microscope to evaluate the percentage that had degranulated.

### 4.7. Detection of Allergen-Specific Antibodies in Serum

The levels of rAmb a 1-specific IgE and IgG2a in the serum were measured as previously described by Qichan Gao et al. [35]. Briefly, 100 ng rAmb a 1 was coated in 96-well plates and the mouse serum was diluted with 1% BSA-PBST (IgG2a, 1:1000; IgE, 1:5; PBST: phosphate buffered saline, 0.05% Tween-20, pH 7.4) and added to the wells (100 μL/well), incubating at 37 °C for one hour. After they had been washed with PBST three times, the HRP-labeled anti-mouse IgG2a, IgE antibodies were diluted with 1% BSA–PBST (1:2000) and added to the wells (100 μL/well), incubating at 37 °C for one hour. After it had been washed with PBST three times, 100 μL TMB reagent was added to develop color and 50 μL, 2M H_2_SO_4_ was added to stop it, the OD value at 450 nm was measured.

### 4.8. Cytokine Determination

Individual spleens were harvested aseptically from each mouse and then cultured in an RPMI-1640 medium containing 10% fetal bovine serum (FBS) in a 37 °C-5%CO_2_ incubator. Subsequently, 20 ug/mL of Ambrosia artemisiifolia crude protein was added to the cells (1.5 × 10^6^ cell/well in 24 well plates) for 72 h and the cell-free supernatant was harvested to detect the IL-4, IL-13, and IFN-γ.

### 4.9. Immunohistochemistry

The sections (4 μm) were incubated with anti-MCP-1 antibody (ab25124, Abcam, America) according to the manufacturer’s instructions. Positive control staining (primary and secondary antibody) and negative control staining (secondary antibody only) were carried out at same time. Then, HRP-goat anti-rabbit IgG antibody (Cell Signaling Technology, Topsfield, MA, USA) was used as a secondary antibody. The results were obtained by staining with DAB reagent and analyzing using a bio-microscope system (Nikon, Tokyo, Japan) and Image-Pro Plus 6.0 software.

### 4.10. Statistical Analysis

GraphPad Prism (version 9.0.0) software was used to analyze the experimental data represented by the mean ± standard deviation. The quantitative data were tested using a Student’s t-test. The *p* value of less than 0.05 indicated that there were significant differences between the groups.

## Data Availability

The data presented in this study are available upon request from the corresponding authors.
